# Analysis of Uncharacterized *mKiaa1211* Expression during Mouse Development and Cardiovascular Morphogenesis

**DOI:** 10.3390/jcdd6020024

**Published:** 2019-06-22

**Authors:** Paige L. Snider, Elizabeth Snider, Olga Simmons, Brenda Lilly, Simon J. Conway

**Affiliations:** 1 HB Wells Center for Pediatric Research, Indiana University School of Medicine, Indianapolis, IN 46202, USA; psnider@iupui.edu (P.L.S.); evsnider@iu.edu (E.S.); osimmons@iupui.edu (O.S.); 2 Biosciences, Indiana University, Bloomington, IN 47405, USA; 3 The Heart Center, Nationwide Children’s Hospital, Columbus, OH 43205, USA; Brenda.lilly@nationwidechildrens.org

**Keywords:** mRNA expression, *mKiaa1211*, *mKiaa1211-like*, mouse heart morphogenesis, neural development, postnatal progenitor tissues, testis, hypomorph

## Abstract

Mammalian *Kiaa1211* and *Kiaa1211-like* are a homologous pair of uncharacterized, highly conserved genes cloned from fetal and adult brain cDNA libraries. Herein we map the *in utero* spatiotemporal expression of *mKiaa1211* and *mKiaa1211L* mRNA and their expression patterns in postnatal testis, skin, gastrointestinal, and adipose progenitor tissues. Significantly, *mKiaa1211* is present throughout the early stages of mouse heart development, particularly in the second heart field (SHF) lineage as it differentiates from mesenchymal cells into cardiomyocytes. We also show that *mKiaa1211* is expressed within several early neuronal tissues destined to give rise to central, peripheral, and sympathetic nervous system structures. Expression profiling revealed that the paralog *mKiaa1211L* is not expressed during the normal developmental process and that *mKiaa1211* expression was noticeably absent from most adult terminally differentiated tissues. Finally, we confirm that a previously uncharacterized CRISPR/CAS-generated *mKiaa1211* mouse mutant allele is hypomorphic.

## 1. Introduction

Even in relatively well-studied model organisms, the majority of coding and both small and long non-coding RNAs have yet to be experimentally characterized [1,2,3]. Establishing a gene’s spatiotemporal expression pattern is an important first step in understanding or predicting the potential physiological and functional role of genes/proteins and how they may interact to form the complex networks that underlie organ formation, function, and homeostasis [4,5]. This is particularly important in developmental biology, since dynamic spatial and temporal gene expression is a fundamental aspect of development [5,6], allied to the rapid and lineage-restricted remodeling events occurring during each morphological stage. For instance, both nervous system and heart morphogenesis are governed by distinct multistep processes that require the coordinated expression of spatiotemporally restricted genes to specify lineage identity and regulate interactions between cells of different origins to complete organogenesis [7,8,9]. Specifically, segmentation of the hindbrain is coupled with the ordered expression of *Hox* genes to generate regional diversity of neural derivatives during craniofacial development [10]; whilst cardiomyocytes destined to populate either the ventricular or atrial chambers express distinct myosin light chains and differentiate in localized regions along the heart tube [11,12]; and progenitor subpharyngeal mesodermal cells outside the early heart (called the second heart field, SHF) undergo delayed differentiation and subsequently contribute to a large part of the definitive heart [13,14].

The remarkable progress in high-throughput whole genome sequencing of multiple species has led to a rapid accumulation in GenBank submissions of ‘hypothetical’, ‘uncharacterized’, or ‘unknown’ genes [15,16]. These predicted genes, which have not been experimentally characterized and whose functions cannot be deduced from simple sequence comparisons alone, now comprise a significant fraction of the public databases [1,2,16]. Assigning functions to the estimated 20,000 protein-coding genes and the ever-expanding number of non-coding RNA genes [3,17,18] is one of the major challenges of the post-genomic era. Indeed, a major bottleneck in genomics is the widening gap between the rapid progress in genome sequencing and the comparatively slow progress in the functional characterization of submitted ‘hypothetical’, ‘uncharacterized’, or ‘unknown’ genes [2]. However, clues to gene function can often be obtained by examining when and where a gene is initially expressed, in which cell lineage/s and/or during whole organism morphogenesis. For example, the Kazusa cDNA project identified ~2000 human genes and many of their mouse counterparts, and these are referred to as the ‘KIAA’ genes [19]. However, many KIAA genes still remain functionally uncharacterized and their biological functions *in vivo* unknown. *KIAA1211* and *KIAA1211-like* are a homologous pair of uncharacterized, highly conserved human genes that were cloned from fetal and adult brain cDNA libraries [20], but their *in utero* onset and spatiotemporal developmental expression patterns are presently unknown.

RIKEN cDNA *C530008M17* (MGI:2444817), also known as *mKiaa1211* in mice, is a relatively unknown gene that was one of six single nucleotide polymorphism targets found to be associated with chicken abdominal fat traits [21] and has recently been demonstrated to be downregulated in colorectal cancer [22]. The human ortholog is *KIAA1211* (HGNC:29219), also called cancer-related regulator of actin dynamics and in adults is thought to act as a tumor suppressor [22]. The paralog of *mKiaa1211* is RIKEN cDNA *2010300C02* (MGI:1919347), which is also known as *KIAA1211L* in humans (HGNC:33454). *mKiaa1211L* is one of several microarray targets associated with patient depression, bipolar disorder, and schizophrenia [23]. Although these limited data suggest *mKiaa1211* and *mKiaa1211L* could have divergent roles, there are no developmental mouse embryo expression data nor a comparison of the spatiotemporal patterns of each paralog. Here we provide detailed expression data at cellular resolution for both uncharacterized genes during mouse development and within specific *in utero* and postnatal progenitor tissues that will help guide future gene molecular, physiological, and functional classification studies. *mKiaa1211* will serve as a useful marker of the SHF during heart morphogenesis.

## 2. Materials and Methods

### 2.1. Quantitative PCR Analysis mKiaa1211 Developmental mRNA Expression Levels

Total RNA was isolated using RNEasy (QIAGEN, Germantown, MD, USA) kit from pooled (*n* = 3 at each stage) C57BL/6N whole embryos, isolated embryo hearts and microdissected cardiac atrial and ventricular chambers (*n* = 3 samples/stage/region). RNA was also isolated from individual newborn and adult organs (*n* = 3 samples/stage/organ). mRNA was reverse transcribed using SuperScript II Reverse Transcriptase and cDNAs amplified within the linear range using two separate pairs of primers. *mKiaa1211* (MGI:2444817) primers were designed to amplify nucleotides 4251–4435 bp and 4452–4586 bp of *Mus musculus* mRNA, and, as both generated similar results (amplified products were sequenced to verify identity), the figures only illustrate 4452–4586 primer data. The following sets of primers were used: 5′ CCACCCATGGCCTTCACATA and 3′ GCATTCAGGAGAGTGAACACCA; 5′ AGGAAAAAGCTGGCTCCCAA and 3′ GTACTGGTAAGACGGGGCAC. *mKiaa1211L* (MGI:1919347) primers were designed to amplify nucleotides 3624–3796 bp of *M. musculus* mRNA using these primers: 5′ GTCCCATCAGAACTTGGCTCA and 3′ CCAGCAGCCTGCCAATCTAT. qPCR was performed in technical triplicate for each sample and qPCR reactions were carried out using SYBR GreenER (Invitrogen, Carlsbad, CA, USA). Loading control and normalization was via GAPDH was as described in [24]. The relative quantification of gene expression between developmental stages, hearts, and/or organs was calculated by the 2^−ΔΔCt^ approximation method. All data are presented as means ± SEM.

### 2.2. Isolation of Mouse mKiaa1211 cDNA Probe

A mouse *mKiaa1211* cDNA probe (designed to amplify part of the *M. musculus* 3′ large fifth exon 4154–4586 bp) was generated via PCR amplification of C57BL/6N adult testes cDNA using established methods [25]. The following primers were used: 5′ TGCCTTGAGCTCCCTCTAGT, 3′ GTACTGGTAAGACGGGGCAC (MGI:2444817) that generated a 433 bp product that was cloned into the pCRII-TOPO vector (Invitrogen, Carlsbad, CA, USA) and sequenced to confirm identity.

### 2.3. In Situ Hybridization and Molecular Marker Immunohistochemistry

Wild type C57BL/6N mouse embryos, microdissected hearts, and isolated newborn and adult organs, as well as *mKiaa1211* mutant mouse samples (see below), were fixed overnight in cold 4% paraformaldehyde, washed in RNA-free phosphate buffered saline, and then either processed for embedding or whole embryo analysis. Following wax embedding and serial sectioning at 10 µm, *in situ* hybridization on at least four to six mouse embryos, serial sections were performed as described in [26]. Similarly, following methanol dehydration and permeabilization, at least three to four whole mouse embryos at each stage were analyzed via whole mount *in situ* hybridization as described in [26]. Both sense and anti-sense non-radioactive RNA probes were synthesized from the cloned *mKiaa1211* cDNA and labeled with digoxigenin using the DIG RNA Labeling kit (Roche, Basel, Switzerland). The specific signal was only observed when sections or whole embryos were hybridized with the anti-sense probe, and serial sections were examined for comparable spatiotemporal patterns of *mKiaa1211* expression in three consecutive serial sections and in at least three individual embryos or organs at each stage of development. Immunostaining of lineage marker monoclonal anti-α smooth muscle actin (αSMA; 1:5000 dilution; Sigma-Aldrich, St. Louis, MO, USA), polyclonal deleted in azoospermia-like (Dazl 1:400; Abcam, Cambridge, MA, USA), polyclonal anti-phospho-Histone (ser10) H3 (pHH3 1:500; Millipore, Burlington, MA, USA), polyclonal hook microtubule-tethering protein-1 (Hook1 1:400; Atlas Antibodies, Bromma, Sweden), and polyclonal anti-Tyrosine hydroxylase (Th 1:5000; Millipore, Burlington, MA, USA) using the ABC kit (Vector, Burlingame, CA, USA) following manufacturer’s directions, was performed on adjacent sections to verify *mKiaa1211* localization and to phenotype mutant mice. 

### 2.4. mKiaa1211 Mutant Mouse Analysis

A CRISPR/CAS-generated *mKiaa1211* mouse mutant allele of the RIKEN cDNA *C530008M17* was generated by the Knockout Mouse Phenotyping Program at The Jackson Laboratory (Bar Harbor, ME, USA; MMRRC Stock #42348-JAX). The alteration resulted in the deletion of 3153 bp, which results in the deletion of exon 6, amino acid change after residue 179, and early termination 15 amino acids later. Heterozygous C57BL/6N-*C530008M17Rik^em1(IMPC)J/^J* mice were intercrossed and the resultant offspring genotyped using either a wild type forward 5′ GAATGTGGTCCCAGTTAAACG or mutant forward 5′ TCCATGGTGATTCTAAGTGCAG PCR primer with a common 3′ GCACAGCAGAGCTTGGAACT primer. Combination of wild type + common primers generated a 136 bp wild type band, whilst mutant + common primers generated a 137 bp mutant band, with heterozygote *mKiaa1211* mutants exhibiting both. Animal procedures and experimental conditions were refined to minimize harm to animals and performed with the approval of the Institutional Animal Care and Use Committee of Indiana University School of Medicine (IACUC#11364 protocol).

### 2.5. Statistical Analysis

The relative quantification of qPCR gene expression between the developmental ages and microdissected organs was calculated by the 2^−ΔΔCt^ approximation method. Three samples per gene from three separate cDNA samples (*n* = 3 developmental stage or organ cDNAs) were obtained to calculate the mean ± SD. Statistical analysis was conducted using the two-tailed unpaired Student’s *t*-test. Differences were considered statistically significant for those with *P* < 0.05. Statistical analysis was performed with Prism software version 5.02 (GraphPad, San Diego, CA, USA).

## 3. Results

### 3.1. Phylogenetic and Comparative mRNA Expression Analysis

**Figure 1:**
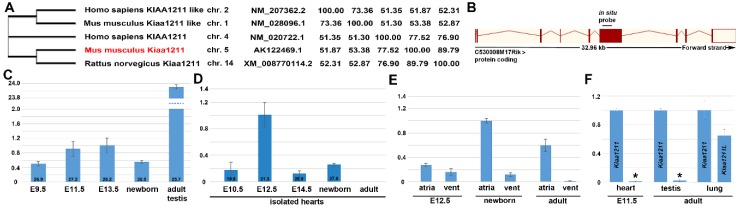
Phylogenetic, genomic structure and comparative qPCR analysis of uncharacterized *mKiaa1211* and *mKiaa1211L* genes. (**A**) Based on NCBI Reference Sequence similarity, *mKiaa1211* (originally called *C530008M17Rik*) and *Kiaa1211L* mouse paralogs, along with human and rat orthologs were aligned and a phylogenetic tree was constructed without distance corrections. Chromosomal locations, NCBI Reference Sequence numbers, and the percent identity matrix (created by Clustal2.1) are shown. (**B**) Ensemble schematic of mouse *mKiaa1211* 6835 bp transcript, with 9 exons (boxes) of 15 total shown. Lines connecting the boxes are introns. Filled boxes are coding sequence, and empty, unfilled boxes are the 3′ untranslated region. The site of the *mKiaa1211 in situ* hybridization probe used in Figure 2, Figure 3, Figure 4 and Figure 5 is indicated. (**C–E**) Quantitative PCR analysis of *mKiaa1211* mRNA levels during development (**C**), in developmentally staged isolated hearts (**D**), and in separate atrial and ventricular heart chambers at E12.5, newborn, and adult stages (**E**). (**F**) Comparison of *mKiaa1211* and *mKiaa1211L* (indicated via *) mRNA expression levels in isolated E11.5 hearts (25.8 vs. 32.4 median cycles) and adult testis (23.6 vs. 31.1 median cycles) but roughly equivalent low levels in adult lungs. qPCR data are presented as a logarithmic plot of relative expression, where a value of 1 indicates no difference between E13.5 whole embryo in (**C**), E12.5 isolated heart in (**D**), and newborn atria in (**E**); and values <1 indicate reduced and >1 indicate increased expression. Error bars represent SD.

Full-length amino acid sequences were used to generate a neighbor–joining consensus phylogenetic tree comparing *mKiaa1211* and *mKiaa1211L* mouse paralogs, along with human and rat orthologs using the neighbor-joining method [27] (Figure 1A). The zebrafish genome also contains orthologues, namely *si:dkeyp-117h8.2* on chromosome 20 (*mKiaa1211*) and *si:ch211-149l1.2* and *si:ch211-121j5.4* (two *mKiaa1211L* variants) on chromosomes 1 and 9. Using the TimeTree tool, *mKiaa1211* has an ortholog in the bacteria *Proteus vulgaris*, indicating the paralog duplicated 4290 million years ago, prior to *mKiaa1211L* [28]. Importantly, alignments (using the BLOSUM62 matrix and the percent identity matrix) demonstrate mouse and rat *Kiaa1211* exhibit greater similarly when compared to human *KIAA1211*, and that *mKiaa1211* and *mKiaa1211L* only have ~51% sequence similarity. Moreover, whilst *mKiaa1211* has 15 coding exons and is on chromosome 5, *mKiaa1211L* has 13 coding exons and is on chromosome 1 (Figure 1B). Thus, it can be seen that across species, *mKiaa1211* and *mKiaa1211L* cluster independently and are distinct coding genes. However, whether these two KIAA paralogs are co-expressed or drive parallel effects, functional divergence, and genetic redundancy is presently unknown.

In order to examine the spatiotemporal mRNA expression patterns of *mKiaa1211* and *mKiaa1211L*, we initially used developmental qPCR analysis. *mKiaa1211* is detectable in embryonic E9.5 whole mouse embryos, with levels increasing during E11.5 and E13.5 stages, followed by a reduction in newborns (Figure 1C). As Mouse ENCODE transcriptome data indicate that the adult testis expresses the highest levels, we used a 12-week-old mouse testis as a positive control and as a gauge of the relative expression levels in mouse embryos. qPCR revealed *mKiaa1211* E11.5 levels are significantly (~24-fold) less than in adult testis. To assess potential *in utero* cardiac expression, isolated microdissected hearts from E10.5–adult stages were examined (Figure 1D). Significantly, *mKiaa1211* mRNA levels were highest around ~E12.5 but scarcely detectable in isolated adult hearts (which is in agreement with Mouse ENCODE transcriptome adult heart data). Isolated hearts were separated into atrial versus ventricular chambers, and *mKiaa1211* expression was examined (Figure 1E). qPCR revealed E12.5 atria and ventricles expressed roughly equivalent levels but both newborn and adult hearts expressed higher relative atrial levels than the ventricles. Examination of the mouse paralog demonstrated that *mKiaa1211L* was absent from isolated E11.5 hearts and testis, but both *mKiaa1211* and *mKiaa1211L* were relatively equivalently expressed at low levels in adult lungs (Figure 1F). Combined, these expression profiling data indicate *mKiaa1211* is the major KIAA paralog expressed *in utero* and is mainly present in embryo hearts and in adult atria and testis.

### 3.2. In Situ Hybridization Analysis of Developmental Spatiotemporal Expression

**Figure 2:**
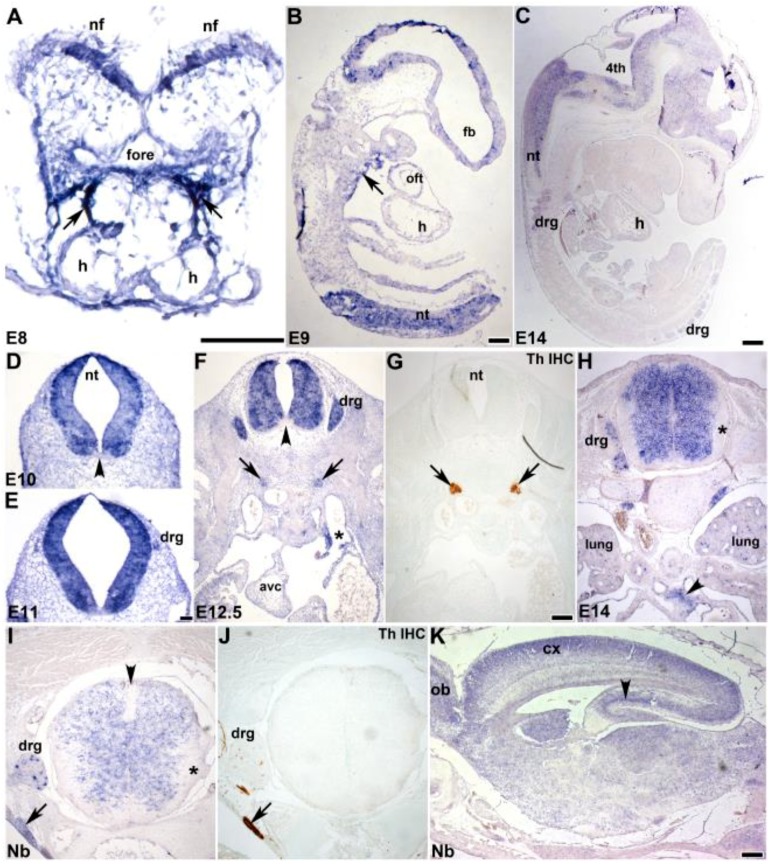
Developmental analysis of *mKiaa1211* expression. (**A**) Non-radioactive *in situ* hybridization detection using digoxigenin (DIG)-labeled *mKiaa1211* antisense cDNA probe on E8 transverse sections revealed mRNA expression (purple/blue stain) in neural folds (nf) and second heart field (SHF)/first branchial arch/outflow tract region (arrows), where the primitive tubular heart attaches to the pharyngeal foregut (fore). (**B**) At E9, *mKiaa1211* is present in sagittal sections in the neural tube (nt) and fore- and hindbrain, as well as in the branchial arch/second heart field region (arrow). (**C**) Similarly, *mKiaa1211* is present in the sagittal E14 section neural tube, hindbrain, and brain, as well as the dorsal root ganglia (drg). (**D–K**) *mKiaa1211* is expressed throughout the transverse E10 (**D**), E11 (**E**), and E12.5 neural tubes (**F**), except in the floor plate (arrowhead (**D**,**F**)); but is lost in the fetal and newborn (Nb) marginal layers (*H,**I**) and roof plate (arrowhead (**I**)) of the central nervous system spinal cord. In newborn sagittal brain, *mKiaa1211* is present in cortex (cx), olfactory bulb (ob), and hippocampus (arrowhead (**K**)). *mKiaa1211* is also expressed as the dorsal root ganglia are formed (E11–birth), and during morphogenesis of tyrosine hydroxylase (TH)-positive ganglia (**G**) of the sympathetic nervous system ((**F**–**J**) arrows). *mKiaa1211* is detectable in the embryonic sinus venosus (* **F**) and fetal vagal (X) trunks (arrowhead (**H**)), along with some punctate expression in E14.5 lung (**H**). Scale bars: (**A**,**B**,**F**–**H**) = 100 µm; (**C**–**E**) = 50 µm; (**K**) = 200 µm. Abbreviations: avc—atrioventricular cushion; 4th—fourth ventricle; fb—forebrain; h—heart; oft—outflow tract; v—heart ventricle.

Non-radioactive *in situ* hybridization was used to confirm the localization of *mKiaa1211* mRNA *in utero.* Both control sense and anti-sense digoxigenin-labeled cDNA probes were transcribed and used for *in situ* hybridization [26]. The specific signal was only observed when sections were hybridized with the anti-sense *mKiaa1211* probe and the same expression pattern was observed in three consecutive serial sections. The earliest detectable *mKiaa1211* was in E8.0 mouse embryos (containing four to six somite pairs), specifically in the neural fold neuroepithelium and adjacent to the foregut endoderm within the SHF/first branchial arch/outflow tract region, where the primitive tubular heart attaches to the pharyngeal foregut (Figure 2A). *mKiaa1211* was also detected in the embryonic sinus venosus, adjacent to the atria (Figure 2F) and punctately in the E14.5 lung (Figure 2H). More-developed E9 embryos and E14 fetuses continued to express *mKiaa1211* in the neural tube, central nervous system, brain, and spinal cord as well as in the peripheral nervous system dorsal root ganglia; and *mKiaa1211* expression was also present in the E9 dorsal wall of the pericardial cavity adjacent to the outflow tract (Figure 2B,C). Detailed analysis of the nervous system, indicated *mKiaa1211* was robustly expressed during the initial formation of the neural folds (Figure 2A), throughout the closed neural tube (except for the floor plate region adjacent to the notochord, Figure 2D–F), and in the mantle layer of the fetal spinal cord (Figure 2H,I). However, in the fetal and newborn spinal cords, *mKiaa1211* was expressed at reduced levels and was only in the mantle layer but absent from the spinal cord marginal layer and roof plate (Figure 2H,I). Within the newborn brain, *mKiaa1211* was most highly expressed in the cortex, olfactory bulb, and hippocampus (Figure 2K). In addition to the central nervous system, *mKiaa1211* was detected throughout the dorsal root ganglia morphogenesis, including their earliest formation ~E11 as primitive bipolar cells, E12.5 transitional bipolar neurons, and maturation as E14–newborn late transitional bipolar and pseudounipolar neurons (Figure 2E,F,H,I). *mKiaa1211* expression was highly localized in a subpopulation of newborn dorsal root ganglia cell bodies, similar to the restricted pattern seen with tyrosine hydroxylase (TH) in developing dorsal root ganglia neurons. [29]. TH is the rate-limiting enzyme of catecholamine biosynthesis and is a definitive marker of the sympathetic ganglia in embryos [30] and is co-expressed with *mKiaa1211* in E12.5–newborn sympathetic ganglia (Figure 2F–J) and fetal vagal (X) nerve trunks that will innervate the neonatal and adult heart (Figure 2H). These expression patterns revealed that *mKiaa1211* was expressed very early in development in multiple progenitor cell tissues and that as development proceeds expression becomes more restricted within the different organ systems.

### 3.3. Cardiovascular Spatiotemporal mKiaa1211 Expression Analysis

**Figure 3:**
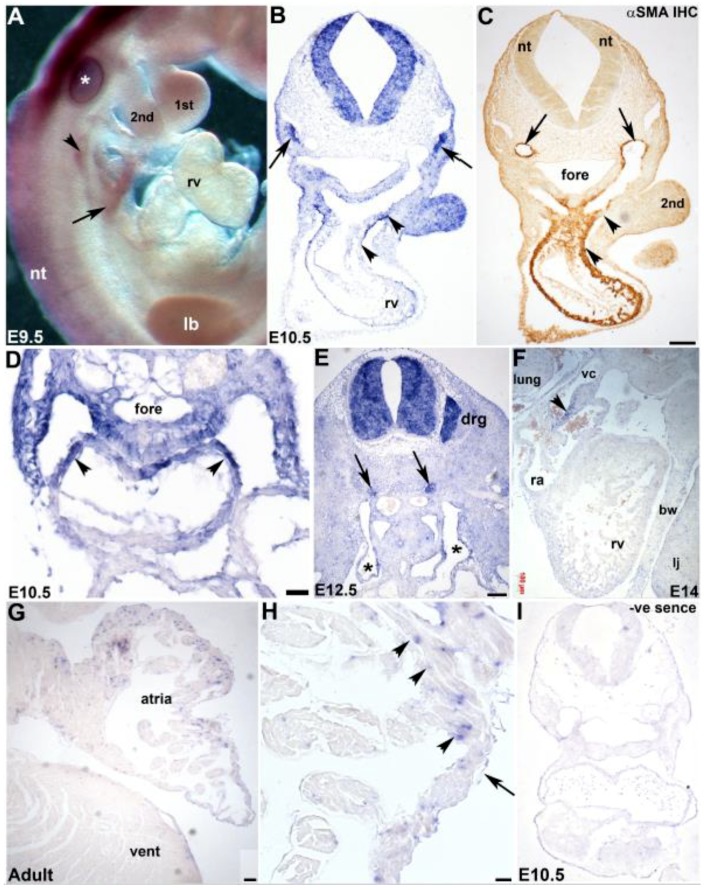
Analysis of *mKiaa1211* cardiovascular expression. (**A**) Whole mount *mKiaa1211* expression is present in E9.5 neural tube (nt), as well as in the SHF (arrow). Additionally, *mKiaa1211* is expressed in glossopharyngeal ganglia (arrowhead). Note staining in otic vesicle (*) is artefactual trapping. (**B**,**C**) Non-radioactive *in situ* hybridization (**B**) and αSMA myocyte marker antibody (**C**) in adjacent transversely sectioned E10.5 embryo. Note *mKiaa1211* and αSMA overlap in the outflow tract/SHF region and right ventricle (rv), specifically in the cardiomyocyte outer layer (arrowheads (**B**,**C**)). *mKiaa1211* is also expressed in the second branchial arch (2^nd^) and in mesenchyme (arrows B) between cardinal veins and αSMA-positive dorsal aorta (arrows (**C**)). (**C**) Whole mount *mKiaa1211* expression is present in neural tube (nt), as well as in the SHF (arrow). Additionally, *mKiaa1211* is expressed in glossopharyngeal ganglia (arrowhead). Note staining in otic vesicle (*) is artefactual trapping. (**D**) *mKiaa1211* is in the E10.5 inflow region, in the walls of the atria (arrowheads) and foregut (fore) endoderm. (**E**,**F**) *mKiaa1211* is in both left and right horns of the E12.5 sinus venosus (* in (**E**)), neural tube, sympathetic ganglia (arrows), and dorsal root ganglia (drg) in transverse sections. Similarly, *mKiaa1211* is in the sagittal E14 wall of the vena cava (vc) just before it enters the right atrium (arrow (**F**)). (**G**,**H**) Adult atrial *mKiaa1211* expression is in a punctate pattern amongst myocytes (arrowheads, (**H**)) but is absent from overlying endothelial lineage (arrow). (**I**) Non-radioactive *in situ* hybridization detection using DIG-labeled (negative control) *mKiaa1211* sense cDNA probe did not reveal any specific expression in E10.5 embryos. Scale bars: (**B**,**C**) = 50 µm; (**D**) = 20 µm; (**E**–**H**) = 100 µm. Abbreviations: 1st—first branchial arch; a—atria; bw—thoracic body wall; lb—limb bud; lj—lower jaw; ra—right atria; rv—right ventricle; vent—ventricle.

As qPCR revealed, *mKiaa1211* was mainly detected *in utero* in the heart and *in situ* hybridization confirmed it was initially present in the SHF/outflow tract of the heart tube, we used both section and whole mount *in situ* hybridization to further characterize which cardiac cell tissues and what chambers express this KIAA paralog. We used whole mount in situ hybridization in younger stages, as this has the advantage of providing whole embryo 3D view of *mKiaa1211* expression. Significantly, *mKiaa1211* was expressed in the E9.5 dorsal pericardial wall that is contiguous with the outflow myocardium and in the pharyngeal region, as well as the sinus venosus and the neural tube (Figure 3A). We also used section *in situ* hybridization for detailed analysis of older ages. In addition to the E10.5 neural tube and second branchial arch mesoderm, *mKiaa1211* was specifically expressed in the heart’s outflow tract outer layer that is composed of cardiomyocytes derived from the SHF [13,14] (Figure 3B). There was continuity of *mKiaa1211* expression from the dorsal wall of the pericardial cavity across the aortic sac into the myocardial wall of the outflow tract and embryonic right ventricle, similar to that observed using the myosin light chain SHF reporter [13]. Moreover, these *mKiaa1211* expressing SHF cells co-expressed the cardiomyocyte differentiation marker α-smooth muscle actin (αSMA) within the outflow tract myocardium (Figure 3C). Serial analysis throughout E10.5 hearts revealed *mKiaa1211* was also expressed in the other pole of the heart, the inflow region, in the walls of the atria and foregut endoderm, and in the atrial chamber and sinus venosus (Figure 3D). At E12.5, *mKiaa1211* was present in both the left and right horns of the sinus venosus and at E14 it was expressed in the wall of the vena cava as it enters the right atrium (Figure 3E,F). As qPCR also revealed, *mKiaa1211* was expressed more robustly in atrial than in ventricular chambers, *in situ* analysis confirmed this postnatal spatial restriction and demonstrated punctate localization amongst the adult atrial myocytes, presumably within the cardiac fibroblast lineage (Figure 3G,H). Combined, these expression analyses demonstrate that *mKiaa1211* was expressed in SHF mesodermal cells that initially reside outside the early heart but undergo delayed differentiation and subsequently colonize both the outflow and inflow poles and contribute to a large part of the definitive heart, including the atria [13,14].

### 3.4. Fetal and Postnatal Spatiotemporal Analysis of mKiaa1211 mRNA

**Figure 4:**
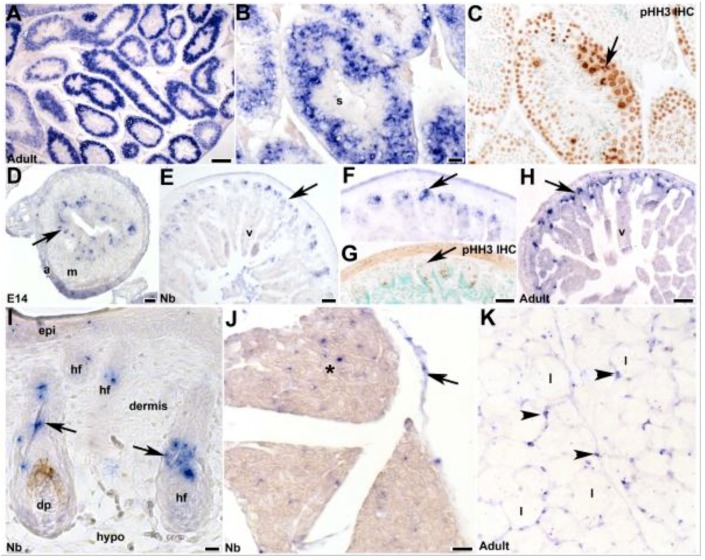
Spatiotemporal analysis of fetal and adult *mKiaa1211* expression. (**A–C**) Non-radioactive *in situ* hybridization in adult testis revealed very robust expression in both mitotic spermatogonia and meiotic primary spermatocytes Type A and B (that contain dispersed chromatin, arrow (**C**)). Proliferating cells were identified using phosphohistone H3 (pHH3) immunohistochemistry on adjacent section (**C**). Spermatids and mature sperm (s) do not express *mKiaa1211*. (**D–H**) *mKiaa1211* is expressed throughout gastrointestinal tract development, with punctate expression present in transverse E14 mesenchymal cells located in close apposition to the endoderm (arrow (**D**), which is the intestinal stem cell niche). In newborn (**E–G**) and adult (**H**) transverse sections, *mKiaa1211* is continually expressed in the crypt stem cell niche (arrow (**E**,**F**,**H**)) prior to their undergoing pHH3+ proliferation (arrow (**G**)). (**I**) *mKiaa1211* is present in fetal, newborn, and adult skin, with limited expression present in abdominal newborn skin epidermis (epi) but robust expression in the root sheath and connective tissue/bulge (stem cell region, arrows (**I**)) surrounding the hair follicles but is absent from the dermis and dermal papilla/hair bulb itself. (**J**,**K**) *mKiaa1211* is also expressed punctately in brown adipose (* in (**J**)) and fibrous septae (arrow (**J**)) and in white adipose tissue nuclei (arrowheads (**K**)). Scale bars: (**A**,**H**) = 100 µm; (**B–D**,**J**) = 20 µm; (**E–G**) = 50 µm, (**I**) = 10 µm. Abbreviations: a—gastrointestinal (GI) adventitia layer; dp—dermal papilla; epi—epithelium; hf—hair follicle; hypo—hypodermis; l—large lipid droplet; m—GI muscularis layer; v—villi.

As Mouse ENCODE transcriptome data indicate that the adult testis expresses the highest levels, we examined which cell types express *mKiaa1211* in order to help understand its potential function. *mKiaa1211* was very robustly expressed in adult testis in a restricted pattern within the mitotic spermatogonia (germ cells) and meiotic primary spermatocytes Type A and B, whereas interphase spermatids and mature sperm did not express *mKiaa1211* (Figure 4A,B). The majority of *mKiaa1211* mRNA was co-localized with phosphohistone H3 (pHH3)-positive cells (Figure 4C), indicating *mKiaa1211* was expressed in the testis in mainly mitotic undifferentiated progenitor cell tissues. Analysis of adult ovaries failed to detect any *mKiaa1211*, indicating that this KIAA paralog was restricted to the male reproductive system. As the human ortholog *KIAA1211* was recently demonstrated to be frequently downregulated in colorectal cancer [22], we examined *mKiaa1211* throughout the gastrointestinal tract development. Similar to its expression in the testis, *mKiaa1211* was expressed punctately in E14 intestinal stem cell niche cells (Figure 4D). Moreover, *mKiaa1211* was continually expressed in the newborn and adult crypt stem cell niche prior to their undergoing pHH3-positive proliferation (Figure 4E–H). Comparable progenitor cell *mKiaa1211* expression was also observed in newborn skin (Figure 4I), specifically expressed in the root sheath and connective tissue/bulge containing the stem cell region but was absent from the differentiated dermis and dermal papilla/hair bulb itself. Similarly, as *mKiaa1211* was one of six single nucleotide polymorphism targets found to be associated with chicken abdominal fat traits [21], we found that it was expressed strongly in white adipose tissue nuclei containing the adipocyte precursor cells (Figure 4K). Given the restriction of *mKiaa1211* to several progenitor and/or precursor cell tissues in various organs, it is likely that *mKiaa1211* expression was suppressed prior to normal differentiation and acquisition of final cell fate.

### 3.5. Analysis of mKiaa1211 Mouse Mutant Phenotype

**Figure 5:**
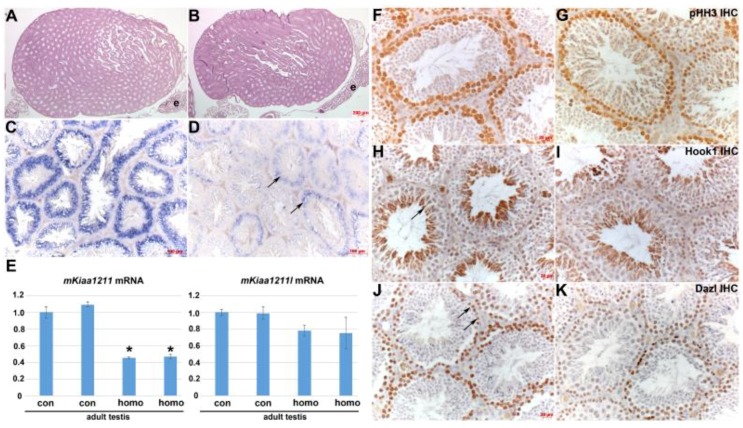
Analysis of *mKiaa1211* mouse mutant phenotype. (**A**,**B**) Using CRISPR-generated *mKiaa1211* mouse mutant (**C530008M17Rik^em1(IMPC)J/^J***)* mice, histology showed that *mKiaa1211/mKiaa1211* homozygous adult testis and epididymis were grossly normal (**B**) when compared to age-matched littermate controls (**A**). (**C**,**D**) Non-radioactive *in situ* hybridization detection of *mKiaa1211* revealed homozygous mutant testis exhibits significantly reduced mRNA levels (arrows (**D**)) when compared to controls (**C**), despite similar patterns of expression. (**E**) Quantitative PCR analysis confirmed that duplicate *mKiaa1211/mKiaa1211* homozygous adult testis express ~60% less *mKiaa1211* mRNA levels (indicated by *) but that *mKiaa1211L* levels are unaffected, when compared to age-matched littermate controls. (**F**,**G**) Homozygous proliferating spermatogonia (identified using pHH3 antibody) were detected in roughly equal numbers. (**H–K**) Immunohistochemistry verified that homozygous mutant (**I**,**K**) and control (arrows (**H**,**J**)) testis express equivalent cytoplasmic Hook1 protein in spermatids, as well as Dazl protein in spermatogonia nucleus. Scale bars: (**A**,**B**) = 200 µm; (**C**,**D**) = 100 µm; (**F–K**) = 20 µm. Abbreviations: e—epididymis.

Using an uncharacterized CRISPR-generated *mKiaa1211* mutant allele mouse model (**C530008M17Rik^em1(IMPC)J/^J***)* generated by the Knockout Mouse Phenotyping Program at The Jackson Laboratory, we examined the functional requirement of *mKiaa1211 in utero* and postnatally. This CRISPR alteration resulted in the deletion of 3153 bp, which results in deletion of exon 6, amino acid change after residue 179, and early termination 15 amino acids later. Heterozygous C57BL/6N **C530008M17Rik^em1(IMPC)J/^J** mice were intercrossed and the resultant offspring genotyped. Surprisingly, we found both male and female homozygous **C530008M17Rik^em1(IMPC)J/^J** offspring at the expected normal Mendelian inheritance rates at 28 days weaning (*n* = 8 L). Moreover, all these homozygotes survived as adults (10–14 weeks) and when homozygous adult male and female littermates were intercrossed, relatively normal litters of homozygous neonatal pups were present (*n* = 5–7 pups/L in 3 L), that still expressed *mKiaa1211* within the SHF cells. Thus, this indicates that the **C530008M17Rik^em1(IMPC)J/^J** allele does not cause any *in utero* lethality or male infertility in 10–14-week-old mice. Moreover, as histology did not reveal any obvious morphological defects in mutant E10.5 embryos and adult testis (Figure 5B), we examined *mKiaa1211* in homozygotes and age-matched littermate C57BL/6N wild type control adult testis (n= 3 of each genotype). *In situ* hybridization revealed that homozygotes expressed significantly less *mKiaa1211* but that the spatial pattern was similar to that of the control testis (Figure 5C vs. Figure 5D). Similarly, qPCR confirmed *mKiaa1211* levels were reduced in homozygous mutant adult testis by roughly 60% but that its paralog *mKiaa1211L* mRNA levels were unaffected in homozygous mutants, when compared to age-matched littermate controls (Figure 5E). Given the observed reduction in *mKiaa1211* levels but normal fertility, we examined whether homozygous mutant proliferating spermatogonia (germ cells) were affected and whether this may have resulted in a secondary effect upon spermatid and mature sperm production (Figure 5F–K). pHH3 immunostaining demonstrated both spermatogonia and meiotic primary spermatocytes were present in roughly equal numbers in homozygotes compared to controls. Similarly, marker analysis confirmed homozygous mutant and control testis expressed roughly equivalent levels of Dazl protein in spermatogonia nucleus as well as cytoplasmic Hook1 protein expression in spermatids. These data indicate that the **C530008M17Rik^em1(IMPC)J/^J** allele is a hypomorphic mutant with a reduction in gene function through reduced mRNA expression but is not a complete loss. Whether testicular expression of the *mKiaa1211L* paralog, which was unaffected in homozygote hypomorphs, is able to compensate for the downregulation of *mKiaa1211* is unknown. However, the relative level of *mKiaa1211L* versus *mKiaa1211* is ~53-fold less (Figure 1F), thus this possibility is unlikely. Therefore, the observed ~60% reduction in *mKiaa1211* adult testis expression levels is not sufficient to aberrantly affect the **C530008M17Rik^em1(IMPC)J/^J** homozygote hypomorphic *in utero* survival or fertility, meaning we were unable to test the functional requirement of *mKiaa1211*.

## 4. Discussion

In this study, we described the spatial and temporal expression of *mKiaa1211* and its paralog *mKiaa1211L* during the normal developmental process and how their expression was affected in a previously uncharacterized hypomorphic *mKiaa1211* mouse mutant allele. Significantly, *mKiaa1211* was present throughout the early stages of mouse heart development, particularly in the SHF lineage that trans-differentiates from mesenchymal cells into cardiomyocytes. Our data also show that *mKiaa1211* was expressed within several early neuronal tissues destined to give rise to central, peripheral, and sympathetic nervous system structures. Expression of *mKiaa1211* was also observed in the neonatal and adult progenitor cell types and was noticeably absent from most adult tissues.

Our results establish that *mKiaa1211* mRNA was first expressed in the E8.0 nascent heart, during the initial stages of SHF specification and during its morphogenesis as they contribute to the outflow tract of the early heart and atrial chambers. Interestingly, *mKiaa1211* was subsequently turned off in fetal and postnatal great vessels exiting the heart but persisted in adult atria within a punctate expression pattern in non-myocyte cells (most likely fibroblasts but could be pericytes, smooth muscle, or immune cells). In fact, many cardiac genes are broadly expressed in the early heart and become restricted to the atria or ventricles as development proceeds [31]. The SHF is a unique pool of cardiac progenitor cells in the early mammalian embryo that initially reside outside the nascent heart but provide a subsequent source of additional myocardium at both the inflow and outflow poles of the definitive heart [13,14]. The SHF is required for normal heart development and when perturbed can often result in congenital heart defects and has also been proposed as a basis for stem cell populations for cardiac repair [32]. Significantly, Wnt/β-catenin signaling is critical for expansion of this SHF progenitor lineage and subsequent normal colonization of the heart [33]. Thus, the recent demonstration that *KIAA1211* expression in adult intestine might be negatively associated with Wnt/β-catenin signaling [22] is intriguing. *KIAA1211* overexpression in human colon cancer cell lines was shown to downregulate Wnt/β-catenin target genes (including the SHF-associated *AXIN2* [34]) and can positively regulate the actin polymerization in vitro in adult intestinal tissue extracts [22]. Together, this may suggest the *mKiaa1211* cardiovascular expression may be Wnt/β-catenin dependent and could help stabilize the SHF progenitor’s actin cytoskeleton once it has differentiated into cardiomyocytes. As many different organ progenitor cells can respond to injury, dedifferentiate, enter the cell cycle, and even self-renew; it will be informative in future to determine whether injured adult hearts re-express *mKiaa1211* and what happens to the *mKiaa1211*-expressing atrial fibroblast population.

Wnt/β-catenin signaling during early vertebrate neural development is also critical, especially for simultaneously establishing cellular diversity and tissue organization [35]. The Wnt/β-catenin pathway also plays a key role in regulating subsequent neural developmental processes, such as patterning along the embryonic axes [35], neural crest emergence and post-migratory differentiation [36,37], and parasympathetic innervation of the heart [38]. As both *mKiaa1211* and *mKiaa1211L* were originally cloned from fetal and adult brain cDNA libraries [20], it is interesting that *mKiaa1211* was initially expressed through the neural folds/tube and as regional speciation and dorsal–ventral and medial–lateral neural tube differentiation occurred. Moreover, *mKiaa1211* became more restricted and was eventually switched off in the adult spinal cord. Similarly, *mKiaa1211* was initially expressed throughout the nascent dorsal root and sympathetic ganglia, but then shut off in adults. Both the *mKiaa1211*-expressing dorsal root and sympathetic ganglia develop in the embryo from neural crest cells, that originally emigrated from the *mKiaa1211*-expressing dorsal neural tube [36,38], but *mKiaa1211* expression was absent in the migratory neural crest phase. This reveals that *mKiaa1211* can be dynamically regulated during embryogenesis, with its expression being switched on and off and on again within the same lineage. Given the developmental expression patterns in both cardiovascular and neural progenitor cells but not in differentiated cell types, it is not unanticipated that *mKiaa1211* would also be expressed postnatally in testis, gastrointestinal, skin, and adipose progenitor cells but not in their derivatives.

In an attempt to examine the functional requirement of *mKiaa1211*, we examined an uncharacterized CRISPR-generated *C530008M17Rik^em1(IMPC)J/^J* mouse allele and surprisingly the homozygote mutants turned out to be hypomorphic with normal fertility and unaffected offspring, despite a ~60% reduction in *mKiaa1211* mRNA levels. Thus, it is unclear whether *mKiaa1211* is a non-essential gene or if the cells that express *mKiaa1211* can function normally with reduced levels. As most genes are inherently multifaceted and complicated via expression in diverse cell types and at different stages, the lack of a phenotype is not always a straightforward outcome. In order to begin to address genetic redundancy, we examined *mKiaa1211’s* paralog *mKiaa1211L* and found that in testis that express the highest levels of *mKiaa1211*, that there was no compensatory upregulation in the hypomorph and further that *mKiaa1211L* was not co-expressed *in utero* in hearts. Interestingly, another CRISPR/CAS-generated *mKiaa1211* mouse allele has been generated, in which the homozygotes exhibit adenoma tumor development in adult small intestine after five to six months of ageing and have pulmonary lesions [22]. In this allele, exon 2 was targeted and immunohistochemistry confirmed the lack of *mKiaa1211* in the adult homozygote intestine. However, it is unclear whether this allele is a null *in utero*, if there is partial *in utero*/neonatal lethality, whether *mKiaa1211* mRNA and protein is absent in all organs, and whether homozygote fertility is aberrantly affected, as these were not the focus of this study. Moreover, it is unclear which *mKiaa1211* antibodies were used to confirm the absence of protein and if they were generated against exon 2 of the *mKiaa1211* protein. Thus, as it remains unclear if there are any observable structural abnormalities and whether a complete null *mKiaa1211* allele would result in an abnormal phenotype, it is difficult to draw definitive conclusions regarding its genetic requirement *in utero*. There are several possibilities why a mouse knockout may result in an overt phenotype: First, the abnormal phenotype is present under the conditions currently being used but is yet to be discovered; second, the abnormal phenotype will only become evident under stress conditions that have not yet been tested; third, there is a functional phenotype which requires more sophisticated expertise; fourth, the gene is part of a large family with closely related structure and physiological function; fifth, there is a subtle phenotype; sixth, there is no abnormal phenotype [39]. Although we did not find paralogous genetic redundancy via *mKiaa1211L*, as *mKiaa1211* is part of a large group of highly conserved human genes [20], it is still possible that structural and/or functional redundancy may could account for a lack of phenotype. Therefore, it remains unclear whether *mKiaa1211* is just a useful informative biomarker or is also functionally required during SHF/progenitor cell morphogenesis which will require definitive genetic testing. Future studies will be needed to further the understanding of this uncharacterized gene (and its paralog) in normal heart and neural development and to ultimately pave the way for the examination of any future potential role in congenital defect pathogenesis and potential use as a basis for stem/progenitor tissue repair and/or regeneration approaches.
